# Supplementary material for: Molecular evidence of pathogens and endosymbionts in the black horse fly Osca lata (Diptera: Tabanidae) in Southern Chile
Source: PLoS Negl Trop Dis. 2024 Sep 27;18(9):e0012525. doi: 10.1371/journal.pntd.0012525 (PMC11463783; doi:10.1371/journal.pntd.0012525)
Supplement: Attachment — Submitted filename: response to the evaluatorsBC - Chile PTND -GE.docx [file pntd.0012525.s001.docx]

Thank you to each reviewer for their time and constructive feedback to improve our manuscripts. Below follows a point-by-point response to their comments and suggestions.

--------------------------------------------------------------------------------------------------------

**Reviewer #1:** (No Response)

**Reviewer #2:** The study's approach seems well-suited to its goals, using molecular methods to determine the prevalence of pathogens in horseflies from chosen areas in Chile. Picking Osca lata and these particular locations makes a lot of sense, especially given how much there still is to learn about this topic in Chile.

1)      The aim/objectives could be improved by:
•       Specifying species used
•       using terms like “prevalence of pathogens”
•       specifying pathogens
•       specifying molecular techniques used

The above has been addressed within the revised version of the manuscript

2)      Is the study design appropriate to address the stated objectives? Yes

3)      The sample size was sufficient given the study being exploratory, n=95.

4)      For clarity add a short sentence as to why nested PCR is used for Rickettsia detection but not for the other pathogens. e.g. increased specificity for Rickettsia detection

Authors. Thank you very much. Added a sentence explaining why the use of semi-nested PCR is necessary

5)      In Table 1, the nested PCR primers for Rickettsia were referenced as "This study." Please clarify if these primers were designed specifically for this study.

Authors. Indeed, the primers used for the semi-nested PCR were designed specifically for this study.

5)      Listing the original names of primers in Table 1 helps others repeat the study and understand where the primers came from. The gltA primers from Labruna et al. were originally named CS-78 etc.

Authors. Thank you very much for your comment. However, in the new analyses performed, the primers of Labruna et al. (2004) were not used, but those of Caspi-Fluger et al. (2012).

6)      L157 is confusing as nested PCR is a specific type of two step PCR rather say conventional and nested PCR.

Authors. Thank you very much for your comment. A sentence was added to clarify what the evaluators pointed out. We believe that the sentence is now easier to understand. L210-213 in the new version of the manuscript.

7)      Please add ethical or regulatory approvals that were obtained for this study

Authors. The code of the ethical approval obtained to conduct the study was added. L161-164 in the new version of the manuscript.

8)      Were sequences obtained from this study submitted to GenBank? The manuscript makes no mention of this or accession numbers retrieved. Please submit to GenBank and add relevant accession numbers for validation of results.

Authors. The sequences obtained in this study were submitted to GenBank. Accession numbers are now included.

**Reviewer #3:** Although the work demonstrates a correct methodology, they do not provide a sample size that is adequate to reach the conclusions they discuss. Having already performed the sequencing, they are not able to reach the species, having used specific primers for such an action. This gives uncertainty and concern in the possible results, since it is different to think of rickettsia rickettsii with r. parkeri, although both share genes, they behave differently, which is why the need to reach the species level for the PCR products that they amplified. So the recommendation would be to sequence with different genes and concatenate to reach genus and species.
Authors. We have conducted an additional lab analysis of our samples to address the concerns you raised. The new sequences are most closely related to an unidentified ***Rickettsia*** endosymbiont of ***Bemisia tabaci***, with 97.7% to 98.3% identity. Therefore, the conclusions in the paper have been modified accordingly.

--------------------

**Results3**
-Does the analysis presented match the analysis plan?
-Are the results clearly and completely presented?
-Are the figures (Tables, Images) of sufficient quality for clarity?

**Reviewer #1:** (No Response)

**Reviewer #2:** Here are a few suggestions:

1) Fig 1-4 are unclear and distorted, I can’t read phylogenetic tree labels. Please add higher quality images. the sample IDs in the figures "23-OTR-51," should explain what they mean and which sample site or species they’re from. Maybe add a SI table to show the information or a short sentence in the methodology section.

Figures 2, 3 and 4 have been enhanced considering journal standards (e.g. 300 dpi for each of the images). GenBank accession numbers have been included for positive samples indicated in the text of the manuscript as, for example, "21-OTRxx.”

2) Bootstrap values are missing from the trees. If they're missing because the support was not significant, it's helpful to mention this in your text to clarify the tree's interpretative value. Authors. We have now ensure that the bootstrap values were added.

3) Subheadings need to be more detailed and precise. For eg. Subheading L145 should add “Sample preparation, DNA extraction and quantification”.
Authors: Thank you very much. The suggested subtitles were included, responding to the comment.

Line 174-185 add sequencing details after PCR and then create a new subheading saying Phylogenetic analysis.

Authors: Thank you. The suggested subtitle was included.

L197-203, It can be confusing to readers the way the percentages and totals are reported rather follow a consistent formatting across all instances where percentages and numbers are [mentioned. E.g](http://mentioned.eg/). Among the 95 samples analyzed by PCR, pathogens were detected in 36.8% (n=35) of the samples. Specifically, Rickettsia spp. DNA was present in 29.4% (n=28) of the samples, Trypanosomatidae DNA in 5.2% (n=5)…. etc
Authors: Thank you. The paragraph was rewritten following the reviewer's indications.

L199-203 can be rewritten for clarity. Eg. In our study, out of the X number of horsefly samples analyzed, two samples (….%) exhibited co-infections with two distinct pathogens. Specifically, one specimen was co-infected with Filarioidea and Rickettsia, while another specimen showed co-infection with….
Authors: Thank you. The paragraph was rewritten following the reviewer's indications.

**Reviewer #3:** The results they show are alarming for public health, since they are focusing on zoonotic pathogens, however, it is necessary to have extracted the DNA from tissue with the ideal kit for tissue and not for blood. The images you show have poor quality, it is necessary to add images with better resolution more than 600 dpi. It is important to keep a table, which can compare which insects presented co-infections.

Authors: Images have been improved in the revised manuscript. Likewise, a table (Table 2) indicating the samples with co-infection is included.

**Conclusions**
-Are the conclusions supported by the data presented?
-Are the limitations of analysis clearly described?
-Do the authors discuss how these data can be helpful to advance our understanding of the topic under study?
-Is public health relevance addressed?

**Reviewer #1**: (No Response)

**Reviewer #2**: The discussion/conclusion section does a great job of shedding light on the intriguing role of *Osca lata* in carrying pathogens in southern Chile. I have a few suggestions/comments for improvement:

1) briefly comparing the pathogens in Osca lata with those in other local vectors like ticks or mosquitoes could give us even more insight into Osca lata's unique role in spreading diseases in the region.

Authors: Thank you. Information on other potential vectors of pathogens in the distribution area of *Osca lata* in Chile was included between L606-623, in the new version of the manuscript. This is evidence of the role of *O. lata* as a vector in southern Chile.

2) Advocate for a "One Health" approach to discuss how understanding the role of Osca lata in pathogen transmission can inform integrated strategies to mitigate the risk of vector-borne diseases.

Authors: Thank you. A paragraph with a "One-Health" approach was included on L627-631, in the new version of the manuscript, to evidence the role of *O. lata* and that integrated strategies be implemented to understand and mitigate the risk of transmission.

Reviewer #3: To reach these conclusions it is necessary to know the biology of insects in comparison to ticks or other potential vectors. Since the feeding process is different in each case. The use of words such as competent and potential is necessary, since demonstrating DNA does not mean that it is capable of transmitting it. It is important to add the points against this theory. There are different works that point to insects as potential vectors, however they mention their biological characteristics.
Authors: Thank you very much for your comment. A paragraph was added, between L642-647 in the new version of the manuscript, to clarify and respond to what the reviewer pointed out.
--------------------

Editorial and Data Presentation Modifications?
Use  this section for editorial suggestions as well as relatively minor modifications of existing data that would enhance clarity. If the only modifications needed are minor and/or editorial, you may wish to recommend “Minor Revision” or “Accept”.

Reviewer #1: (No Response)
Reviewer #2: (No Response)
Reviewer #3: (No Response)

--------------------

**Summary and General Comments**
Use  this section to provide overall comments, discuss strengths/weaknesses of the study, novelty, significance, general execution and scholarship. You may also include additional comments for the author, including concerns about dual publication, research ethics, or publication ethics. If requesting major revision, please articulate the new experiments that are needed.

**Reviewer #1**: This manuscript by Gonzalez et al. reports on the molecular detection of several types of microbes in tabanid horse flies (Osca lata) collected from two regions in Chile. 95 specimens were dissected and mouthparts and guts were tested by PCR using primers that target Rickettsiae, Trypanosomatidae, and Filarioidea. The authors report detection of each of these taxa at appreciable prevalence, which comprises the first study of this type in horse flies from Chile and of this particular horse fly species in general.

The manuscript is overall well-written, and the methods used are appropriate. However, it is my opinion that the manuscript requires some significant revisions before publication. In particular, some aspects of the introduction and discussion require more nuance/detail. Further, there are some issues surrounding the interpretation of the results with regards to the microbes detected being classified as “pathogens” from insufficient or contradictory evidence., and the broad use of the term “pathogen” inappropriately.

Specific points are provided below.

Line 1: I find the title a bit misleading as this manuscript does not directly assess the vector role of horse flies in any way. What is presented here is molecular surveillance/molecular detection of pathogens in horse flies. I would recommend to modify the title slightly by removing the reference to a vector role. Maybe something like “Molecular evidence of pathogens in the black horse fly, Osca lata, from southern Chile”, or something similar.
Authors. Given the comments of Reviewer 1, the title of the paper has been modified.

Line 26: Please check the manuscript throughout for consistent use of the term “horse fly/horse flies”. As these are true flies, it should be two words, but in various parts of the manuscript it is written as one word incorrectly.

Authors. Thank you very much for the correction. The entire manuscript was checked and the word "horsefly" or “horseflies” was corrected to “horse fly” or "horse flies".

Line 26-35: The authors make broad use of the term “pathogen” without evidence in the abstract and throughout the manuscript. While Dirofilaria immitis, which the authors detected, is a pathogen, the other sequences the authors detected do not appear to correspond to pathogens. Additional comments about this are given below, but the authors need to be more discerning when referring to D. immitis vs the other microbes that they detected throughout the manuscript and be clearer that the others do not appear to be pathogens.
Authors. Authors. Thank you very much for your comment. The entire manuscript was revised, and the term "pathogen" was changed to "microorganisms" where appropriate.

Introduction lines 88-102: I found the introduction with regards to the vector role of horse flies to be thin. In would be very useful and improve the manuscript if the authors expanded and gave more details about the types of pathogens that horse flies have been found to vector and the types of pathogens that have been detected in association with them in other regions.
Authors. Thank you very much for your comment. We have added a paragraph, between L113-125, in the new version of the manuscript, to respond to the reviewer's request. We believe that the impact of horse flies, as mechanical vectors, has been much better demonstrated and with explicit mention of pathogens transmitted by them.

Similar to my comment above, information about Osca lata in particular is lacking in the introduction. What is the importance of this species more specifically? Why study it? What, if any, studies have been done in this species? Some of this is mentioned in the discussion but I think should also be in the introduction.
Authors. Thank you very much for your comment. We have added a paragraph in the intro, between L135-147, in the new version of the manuscript, to respond to the reviewer's request. We have provided more background on the distribution range of *Osca lata* and the impact, not evaluated, that it generates on livestock and tourism in the area where this study was developed. Now with our results, we hope to contribute to other studies that contribute to a more comprehensive knowledge of this species.

Line 167: 390 bp product for Rickettsia is relatively small. Although the authors sequenced PCR products for further analysis, they themselves acknowledge that sequencing of this region is inadequate for species level identification. Therefore, related to my comment about the use of the term “pathogen” above, the authors should not refer to this is a pathogen. Indeed, from lines 205 of the results and lines 344 of the discussion, it appears that the sequence detected most closely matched the non-pathogenic Rickettsia, R. bellii.
Authors. Additional molecular analysis was performed. As indicated in L223 of the new corrected version of the manuscript, 760 bp products were available in the new analysis. This allowed us to determine that it is an endosymbiont of different species of Arthropoda. This is in agreement with what is indicated in the "Results" and in the "Discussion".

Line 226: Similar to my point above, Crithidia dedva is an insect specific Trypanosomatid and not a pathogen. Please be more discerning when referring to this microbe and do not refer to it as a pathogen.

Authors. Thank you very much for your comment. Indeed, as the reviewer *Crithidia dedva* points out, it is a species associated with Arthropoda and described in a species of Diptera Sarcophagidae. However, it is interesting to point out, as it is done between L513-523 of the new version of the corrected manuscript, that there are reports in the literature of detection of *Crithidia* in immunocompromised individuals. Therefore, we consider relevant the finding detected in the species studied.

**Reviewer #2:** The study provides valuable preliminary insights into the prevalence of pathogens in Osca lata in southern Chile. Below are suggestions/comments based on abstract and introduction sections. I have included comments for the discussion under the conclusion section.

Abstract

4) "provides evidence of the molecular detection of parasites." , "parasites" is broad, specifically state which parasites.

Authors. We have changed the use of "pathogen" to "microorganism" where appropriate and refer only to *Dirofilaria immitis* as "pathogen".

Introduction:
L113-115 should be removed from the intro as it states study results.

Authors. The requested text was removed and a new paragraph is presented between L154-157 of the new version of the corrected manuscript.

L50-64 seems overdrawn and doesn’t directly relate to your study’s focus. Shorten it and directly introduce horseflies to the first paragraph.

Authors. Part of the paragraph was deleted. The new paragraph is displayed between L70-73 of the new corrected version of the manuscript. We also consider it relevant to contextualize with background information on the hematophagous habit.

In your introduction, several statements are left hanging without any references, for eg. L59-64, L78-82 etc.
Authors. Thank you very much for your comment. References have been included in the paragraph indicated by the reviewer.

**Reviewer #3:** It is necessary to amplify other genes to reach the species of each pathogen they indicate. based on the results obtained from sequencing. They should change or enrich the discussion of their work, since there are different species that can cause acute or subclinical diseases. Add the biology of insects describing the pros and cons of acting as a vector for certain pathogens. Use the terms potential and competent, differentiating which one your hypothesis falls into.
Authors. Thank you very much for your comment. A new paragraph, L642-647, was added to the corrected version of the manuscript to clarify what the reviewer pointed out. We believe it provides a better understanding of the *O. lata* factor in the study area and its potential as a vector.
--------------------

PLOS authors have the option to publish the peer review history of their article (<https://journals.plos.org/plosntds/s/editorial-and-peer-review-process#loc-peer-review-history>). If published, this will include your full peer review and any attached files.

If you choose “no”, your identity will remain anonymous but your review may still be made public.
Do you want your identity to be public for this peer review? For information about this choice, including consent withdrawal, please see https://www.plos.org/privacy-policy

Reviewer #1: No
Reviewer #2: No
Reviewer #3: No
