# Supplementary material for: Molecular evidence of pathogens and endosymbionts in the black horse fly Osca lata (Diptera: Tabanidae) in Southern Chile
Source: PLoS Negl Trop Dis. 2024 Sep 27;18(9):e0012525. doi: 10.1371/journal.pntd.0012525 (PMC11463783; doi:10.1371/journal.pntd.0012525)
Supplement: Attachment — Submitted filename: Response to evaluators 2_ Sept2024.docx [file pntd.0012525.s002.docx]

September 5, 2024

Dear Editor and reviewers,

Thank you very much for your work and time in reviewing and editing our manuscript “**Molecular evidence of pathogens and endosymbionts in the black horse fly *Osca lata* (Diptera: Tabanidae) in Southern Chile**”.

Your comments and suggestions have certainly substantially improved our manuscript.

All suggestions made by each reviewer and the editor have been accepted and incorporated in the re-submission of our manuscript, which we present here as both a “tracked-change” and a “clean” version. We also addressed the clarity of writing in general.

Thank you very much for your consideration.

Gillian Eastwood

Christian R. González
